# Immediate and Short-Term Effects of Upper Cervical High-Velocity, Low-Amplitude Manipulation on Standing Postural Control and Cervical Mobility in Chronic Nonspecific Neck Pain: A Randomized Controlled Trial

**DOI:** 10.3390/jcm9082580

**Published:** 2020-08-10

**Authors:** Francisco Gómez, Pablo Escribá, Jesús Oliva-Pascual-Vaca, Roberto Méndez-Sánchez, Ana Silvia Puente-González

**Affiliations:** 1 Centro Fisioterapia y Osteopatía Osteofisio, 12006 Castellón, Spain; osteofisiocastellon@gmail.com; 2 Clínica Fisioterapia y Osteopatia Pablo Escribá, Alboraya, 46120 Valencia, Spain; pabesast@gmail.com; 3 Department of Physiotherapy, Universidad de Sevilla, 41009 Sevilla, Spain; joliva5@us.es; 4 Department of Physiotherapy, University School of Osuna, Universidad de Sevilla, Osuna, 41640 Sevilla, Spain; 5 Department of Nursing and Physiotherapy, Universidad de Salamanca, 37007 Salamanca, Spain; silviapugo@usal.es

**Keywords:** upper cervical spine, chronic neck pain, musculoskeletal disorders, HVLA manipulation, cervical range of motion, postural balance

## Abstract

This study aimed to determine the immediate and short-term effects of a single upper cervical high-velocity, low-amplitude (HVLA) manipulation on standing postural control and cervical mobility in chronic nonspecific neck pain (CNSNP). A double-blinded, randomized placebo-controlled trial was performed. Forty-four patients with CNSNP were allocated to the experimental group (*n* = 22) or control group (*n* = 22). All participants were assessed before and immediately after the intervention, with a follow-up on the 7th and 15th days. In each evaluation, we assessed global and specific stabilometric parameters to analyze standing postural balance and performed the cervical flexion-rotation test (CFRT) to analyze upper cervical mobility. We obtained statistically significant differences, with a large effect size, in the limited cervical rotation and global stabilometric parameters. Upper cervical HVLA manipulation produced an improvement in the global stabilometric parameters, significantly decreasing the mean values of velocity, surface, path length, and pressure in all assessments (*p* < 0.001; ƞ 2 *p* = 0.323–0.856), as well as significantly decreasing the surface length ratio (L/S) on the 7th (−0.219 1/mm; *p* = 0.008; 95% confidence interval (CI): 0.042–0.395) and 15th days (−0.447 1/mm; *p* < 0.001; 95% CI: 0.265–0.629). Limited cervical rotation values increased significantly immediately after manipulation (7.409°; *p* < 0.001; 95% CI: 6.131–8.687) and were maintained during follow-up (*p* < 0.001). These results show that a single upper cervical HVLA manipulation produces an improvement in standing postural control and increases the rotational range of motion (ROM) in the upper cervical spine in patients with CNSNP.

## 1. Introduction

Neck pain can be defined as “an unpleasant sensory and emotional experience associated with actual or potential tissue damage in the neck region” [1]. It is a common problem that an estimated 67% of the population will suffer from at some moment in their life [2]. The most frequent form of clinical presentation is nonspecific or mechanical neck pain [3]. Some of these patients, after the first episode of cervical pain, will experience a new episode in the following years, most of them in the first year [3], and some of them will not experience complete resolution of pain and disability, which can become a more complex chronic pain syndrome [4].

Chronic nonspecific neck pain (CNSNP) is a neck pain not due to organic pathology or that it has an unknown pathological basis as the underlying cause over the last 12 weeks or more, and is associated with a high economic cost for health [3,4,5], becoming a socio-health problem, since it is associated with disability, muscular alterations, decreased cervical range of motion (CROM) and greater sensitivity to pain [3]. It also relates to alterations of kinesthesia and the proprioceptive system, like postural stability disturbance and joint position sense deficit [6,7,8], according to a systematic review where nonspecific neck pain and the whiplash associated disorder exhibit postural instability with greater center of pressure (COP) excursions [9]. It has also been proven that experimental neck muscle pain significantly impairs standing balance, suggesting the need for intact neck neuromuscular function for the maintenance of a standing posture [10].

Proprioception is the interoceptive sense that includes kinesthesia (movement sense) and the position sense (joint position sense) [11,12]. It is also essential for effectual sensorimotor control, with important roles in feed-back and feed-forward control [13]. The cervical sensory afferents are involved in balance control and have relatively strong influences on the vestibular and visual system [13,14]. It is also known that muscle spindles are major kinesthetic proprioceptors, with a very high density, specifically in the suboccipital region [15].

Some studies have shown evidence of the benefit of manual therapy, observing that is possible to enhance kinesthetic sensitivity and proprioception [16]. Different effects have been observed after high-velocity, low-amplitude (HVLA) manipulation in different regions, such as peripheral joints [17,18], sacroiliac joints [19,20], and the spine [21,22], both on pain and functionality [21,22,23,24], as well as neurophysiological effects on cortical somatosensory integration and function due to transient cortical plastic changes [25], on the neuromuscular system [23,25,26], kinesthesia [21], and global postural control [26]. The neurophysiological effects after HVLA manipulation seem to be proven, although, the clinical relevance, in relation to the neurophysiological changes, is not completely known [23]. For this, as the systematic review by Honoré et al. indicates, it would be important to review the previous studies considering the effect size, not only in regard to the immediate effects, but also the medium and long-term effects [27] because usual treatments for chronic neck pain achieve at most moderate effects in the short-term [28].

The last Clinical Practice Guideline by the American Physical Therapy Association recommended the use of cervical HVLA manipulation for the management of neck pain guided by impairment and function related assessment of the cervical spine [29]. In addition, some studies reported positive results on load distribution and postural control after HVLA manipulation in different regions [17,18,19,20,30,31], while other studies showed low or no clinical effects [32,33].

Patients with mechanical neck pain have C1-C2 rotational mobility restriction [34], and we know that cervical rotation affects standing postural control [35], considering that muscle spindles are major kinesthetic proprioceptors, with a very high density, specifically in the suboccipital region [15].

Thus, the purpose of this randomized clinical trial was to examine the acute and short-term effects of a single upper cervical spine (C1-C2) HVLA rotational thrust manipulation on CROM and standing postural control in patients with CNSNP.

## 2. Materials and Methods

### 2.1. Study Design

A randomized, double blinded controlled trial, with two arms (experimental and control), was conducted to evaluate the acute and short-term effects of the intervention. The experimental group received a single upper cervical spine (C1-C2) HVLA rotational thrust manipulation, while the control group received a sham technique. There were four assessments, at baseline, immediately after the intervention, and on the 7th and 15th days after the intervention. The study protocol complied with the ethical guidelines of research involving human subjects, was conducted according to the Helsinki Declaration, was previously approved by the Institutional Ethics Committee, and was prospectively registered in the Australian New Zealand Clinical Trials Registry, anzctr.org.au (ACTRN12615000214538).

### 2.2. Sample Size Calculation

The sample size was based on a preliminary pilot study (12 subjects), using the free software “GRANMO simple size calculator” (7.12 version) and considering the surface length ratio (L/S) (1/mm), also called the length in function of surface (LFS), for the calculation, since it was the most demanding primary outcome in the calculation. L/S is the ratio of stroke length and the surface of the ellipse, and provides information about the accuracy of standing postural control and the energy cost or effort made by the subject. When L/S values decrease, subjects find maintaining the standing position harder, and the postural system requires more effort [36]. We determined that accepting an alpha risk of 0.05 and a beta risk of 0.2 in a bilateral contrast, a minimum of 22 subjects will be required in the first group and 22 in the second to recognize as statistically significant a difference equal or greater than 0.44 (1/mm). It was assumed that the common standard deviation is 0.4 and a drop-out rate of 10% was anticipated.

### 2.3. Participants and Recruitment

**Figure 1:**
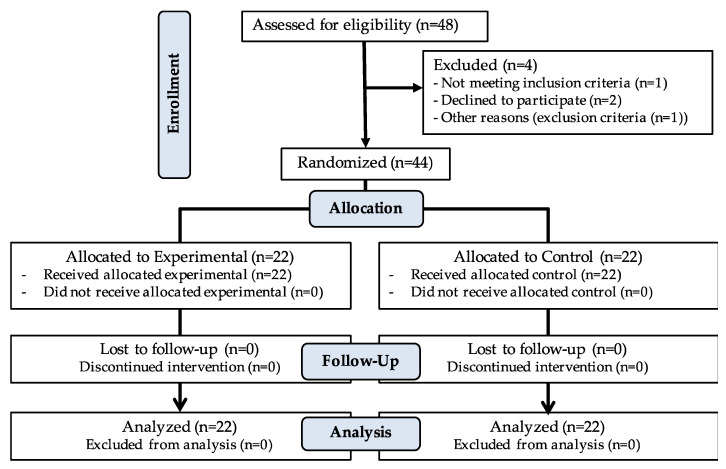
CONSORT flow diagram.

In total, 44 patients were included in the study after selection criteria who voluntarily signed the informed consent (4 patients were excluded in the enrollment) (Figure 1). Patients were recruited consecutively in a private clinic (21 men and 23 women). They were between 18 and 60 years old, had CNSNP (over 12 weeks or more) higher than 2 in an 11-point Numeric Pain Rating Scale (NPRS) [37], with positive CFRT in one side (below 32–33 degrees) [38,39], and without any contraindication to cervical manipulation. Exclusion criteria were (a) vascular, neurological, or rheumatic pathology, or congenital malformations in the head or cervical spine; (b) alterations that affect the balance or postural control (e.g., dizziness, vertebrobasilar insufficiency, or Klein test positive); (c) traumatic antecedents on head or spine (e.g., fractures, sprains, whiplash); (e) pain in regions other than the neck or head during the assessments; (f) drug treatment (e.g., muscle relaxant, analgesic, anti-inflammatory).

### 2.4. Masking and Allocation

We performed a double-blinded study, where the patients and the assessor were blinded. The patients did not know which were the interventions that were being compared. Further, the patients in the control group did not know that the ultrasound was turned off. In regard to the assessor, he did not know anything about the intervention that every patient had received. Thus, he did not know which group they belonged to. After the baseline assessment, the physiotherapist that applied the intervention performed the allocation. The randomization was undertaken using a computerized randomization system (randomized.com), and allocation concealment was guaranteed by sequentially numbered, opaque, sealed envelopes. An outside coworker safeguarded the sequence for those participating in the study.

### 2.5. Outcome Measures

We considered CFRT and L/S as primary outcomes, which were used as inclusion criteria in the recruitment and selection of subjects and for the calculation of the sample size, respectively.

#### 2.5.1. Cervical Flexion-Rotation Test (CFRT)

The CFRT is a commonly and easily applied assessment to determine the presence of a joint dysfunction at the C1-C2 level, and has been reported to be a reliable and valid measurement of upper cervical movement [40,41]. It has shown excellent inter-tester reliability (ICC 0.93 (confidence interval (CI), 0.87–0.96)) and examiner agreement of its interpretation (κ value 0.85). The validity to identify C1-C2 dysfunction also excellent (sensitivity 90%; specificity 88%) with a likelihood of a correct diagnosis of 89% [40]. Subjects lay down in supine position with the CROM^®^ instrument on the head (Platismo Airguide Inc Compas, Buffalo Groove, IL, USA) [39,40]. The evaluator performed a maximum range of cervical flexion in an attempt to block, as much as possible, above and below C1-C2. Then, the evaluator rotated the head to each side like Hall et al. described [38,40,42]. The mean of three measurements of each side was used for further analysis.

The test is positive when there is a limitation of movement below 32–33° (10° less than the movement considered normal) [38,39,40,41,42,43]. The limitation is determined when resistance or pain appears in the cranio-cervical region when performing head rotation [43].

#### 2.5.2. Static Postural Stability Assessment

Postural assessment was examined with the subjects standing on a force platform (NAMROL PODOPRINT, Software analysis PODOPRINT 2.9V, Medicapteurs, France, SAS) with the recommendations of the systematic review published by Ruhe A. et al. to achieve acceptable reliability for most COP parameters [44]. Data were sampled at 100 Hz, over 90 s, in the standing position, and different parameters of COP excursions were analyzed (displacements, velocities, areas and pressures) [44,45,46]. A positioner for the feet was used considering, as reference, the tubercle of the scaphoid, leaving a separation of 5 cm between the feet. Eyes were closed and the jaw was relaxed without occlusal contact. Before starting the measurement, the subjects were instructed to “be as still as possible with eyes closed, breathe normally, do not talk, do not tighten the jaw, and hold that position until I tell you” [18,20,44].

### 2.6. Interventions

The therapist that applied the interventions in both groups was an experienced physiotherapist and osteopath with more than 13 years experience treating patients suffering from neck pain by means of manipulative technics. After baseline assessment and performing the allocation, the subjects in both groups were treated with a different intervention for only one session.

#### 2.6.1. Experimental Group

**Figure 2:**
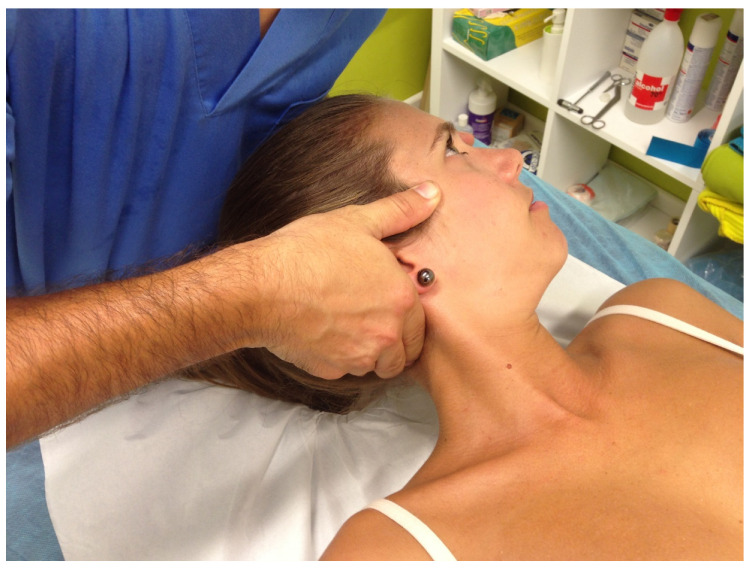
Upper cervical spine (C1-C2) high-velocity, low-amplitude (HVLA) rotational thrust manipulation for a restricted left rotation with positive cervical flexion-rotation test (CFRT).

A single upper cervical spine (C1-C2) HVLA rotational thrust manipulation was applied with the patient in the supine position. The rotational thrust was applied to the side that was positive in the CFRT. For a restricted left rotation, the physiotherapist was in contact with the right posterior arch of the atlas with the proximal index phalanx of the right hand and the left hand cradled the head of the patient. To localize the tension to the C1-C2 level secondary (posterior-anterior shift on the right posterior arch of the atlas and left side-shift) and primary (left rotation and right side-bend), levers of movements were used. Then, the physiotherapist applied a thrust in left rotation in an arc toward the underside eye and translation toward the table [24,34,47] (Figure 2). The patient remained on the table until an alarm went off, indicating when the technique had finished.

#### 2.6.2. Control Group

The sham technique consisted of the physiotherapist placing his hands on the patient’s neck, without adding pressure or therapeutic intention, while a turned-off ultrasound was performed without movement of the head, simulating static application of the ultrasound, applied for one minute. At the end of the minute, an alarm went off, indicating that the time had finished.

### 2.7. Statistical Analysis

Statistical analysis using IBM SPSS Statistics (v.23) and R Statistical Package (v.3.0.1) was performed (http://cran.r-project.org). Descriptive analysis of demographic and clinical characteristics was performed to summarize the data, including frequency for categorical variables and measures of central tendency and dispersion for continuous variables. Baseline data were compared between the manipulation group and control group using independent t tests for continuous variables, as well as chi square tests of independence for categorical variables.

Two-way repeated measures ANOVA was performed (group * time). Group factor was considered as a categorical variable in the statistical model and time factor was used by taking into account the baseline and the three post-intervention data (immediate, 7th, and 15th days). The interaction between the two factors on the dependent variables was analyzed. Regardless of whether there is an interaction, follow-up tests (post-hoc) were performed to determine how the within-subjects factors affected the dependent variables. If a baseline value was found to be significantly different considering the baseline values, it was used as a covariate in the model. The post hoc Sidak test to determine which pairs of means have significant differences was calculated.

Analysis of whether the values of two or more quantitative variables changed in conjunction was performed by correlations with Pearson’s r coefficient.

The level of significance for the statistical tests was set at *p* ≤ 0.05 with the confidence interval of 95%.

In order to assess the magnitude of the change in the result variables, the effect size of the upper cervical manipulation for the group * time ANOVA model was calculated as the partial eta squared (ƞ 2 *p*) when significant, considering 0.01 small, 0.06 medium, and more than 0.14 a large effect size [48].

## 3. Results

### 3.1. Descriptive Analysis

Independent variables such us age, gender and anthropometric parameters (weight, height, and body mass index (BMI)), did not present significant differences between the experimental and the control group in baseline data. At that moment, we did not find significant differences before the intervention comparing the values of dependent variables between groups, except in the limited rotation, where, even finding differences between both groups, the values were within the data sought in the CFRT as positive (less than 32–33°), so that both groups were considered homogeneous. Table 1 shows the characteristics of each group at the beginning of the study.

### 3.2. Inferential Analysis

In the inferential analysis with two-way repeated measures ANOVA (Table 2), statistically significant differences were obtained for the group factor (regardless of time) with *p* < 0.01 in the limited rotation, mean velocity, mean path length, and mean COP-Y, and with *p* = 0.017 in L/S (Table 2). In the interaction between factors (group * time), we observed significant differences, with *p* < 0.01 in limited rotation and global stabilometric variables and with *p* < 0.05 in some COP specific displacements variables (mean COP-Y, anterior, and lateral velocity).

The effect size achieved with the intervention in the experimental group over time, compared with the control group, showed a large effect, especially in the limited cervical rotation and in the global stabilometric variables of COP displacement, being ƞ 2 *p* > 0.5 in all of them (Table 2).

To analyze in more detail, the significant interaction between factors (group * time), we applied the post hoc Sidak test to determine which pairs of means have significant differences. We analyzed the comparisons between subjects from different groups in each evaluation and we also analyzed intragroup comparisons for the time factor (baseline, immediately after intervention, and 7th and 15th days).

#### 3.2.1. Inter-Group Comparisons

**Figure 3:**
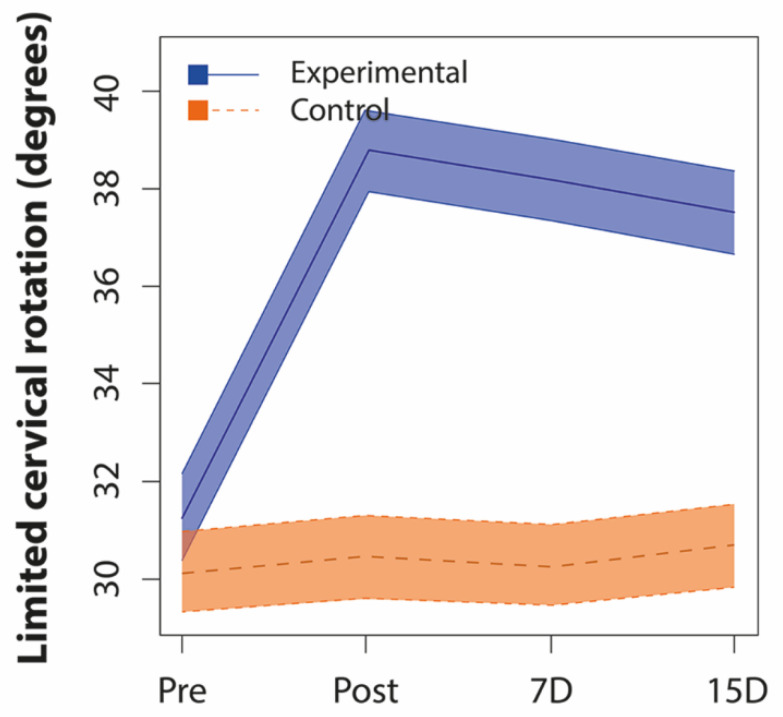
Differences with 95% confidence interval in behavior during intra-subject follow-up of limited cervical rotation between both groups. Pre: Pre-intervention baseline data; Post: Post-immediate data; 7D: Data on the 7th day; 15D: Data on the 15th day.

**Figure 4:**
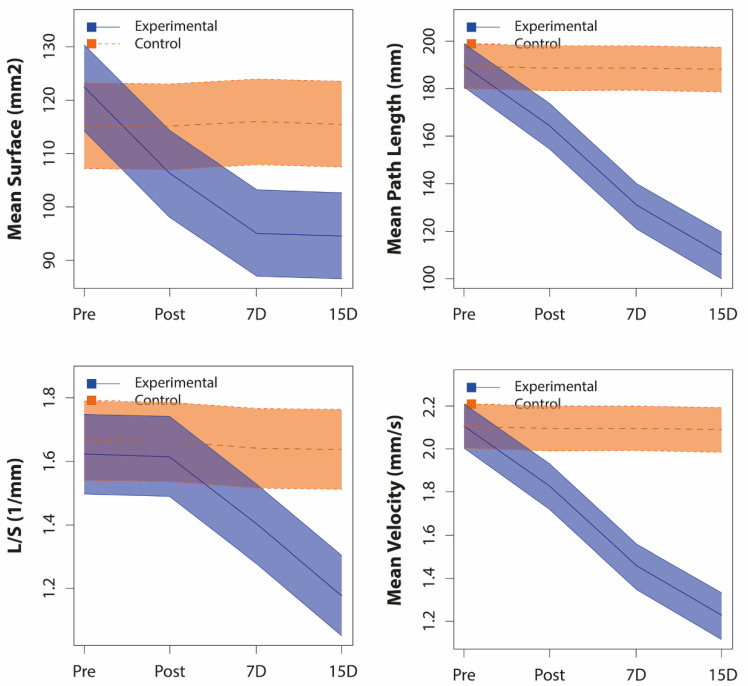
Differences with 95% confidence interval in behavior during intra-subject follow-up of global stabilometric variables between both groups. L/S: Length in function of the surface; Pre: Pre-intervention baseline data; Post: Post-immediate data; 7D: Data on the 7th day; 15D: Data on the 15th day.

Limited cervical rotation showed significant differences between groups in all evaluations (*p* < 0.01) (baseline differences already considered) (Figure 3), with a much higher mean value (between 6.8 and 8.3 degrees) with respect to the control group in three post-intervention evaluations (Table 3). In global stabilometric variables, similar values could be observed in all of them (Figure 4), except for the mean pressure, where no significant differences were obtained between groups in any of the evaluations, although a different intra-subject behavior was observed in both groups. In mean velocity, mean surface, and mean path length, the differences between groups began in the post-immediate evaluation and remained until the end of the study (*p* < 0.01), while, in the L/S ratio, the differences were observed from the evaluation on the 7th day (*p* < 0.01). In COP-specific displacements variables, we only observed significant differences between groups in mean COP-Y, from the post-immediate evaluation (*p* < 0.01) (Table 3).

#### 3.2.2. Intra-Group Comparisons

In intra-group comparisons, there were no significant differences for the control group between the different evaluations in any of the variables studied. Meanwhile, the behavior of the experimental group showed evident changes during the study, with statistically significant differences in all the variables studied, except in mean COP-X (Table 4), which could explain the significant size of the effect achieved by the intervention studied in the analysis using the partial eta squared (ƞ 2 *p*).

In the intra-group comparisons for the experimental group (Table 4), an immediate increase in limited cervical rotation was observed (7.409 ± 0.463 degrees; *p* < 0.01), which was maintained throughout the follow-up until the end of the study. In the primary outcome variable L/S, we observed that the significant changes did not appear until the 7th day, with a variation of 0.219 ± 0.064 1/mm (*p* < 0.01). In the remaining global stabilometric variables (velocity, surface, path length, and pressure), we observed a significant change since the post-immediate evaluation (*p* < 0.01). In COP-specific displacements variables (mean COP-Y and mean anterior velocity), changes were also obtained from the post-immediate evaluation (*p* < 0.05), while, in mean lateral velocity, a significant change in the evaluation was only observed on the 15th day compared to the baseline data of the study (*p* < 0.05).

#### 3.2.3. Correlations between Limited Cervical Rotation and Stabilometrics Variables

In the current study, we were interested in analyzing the correlations between limited cervical rotation and stabilometric variables. In the baseline data, there were no significant correlations between limited cervical rotation and global or specific stabilometric variables (*p* > 0.05), except for the correlation with the mean COP-Y (ρ = −0.339; *p* = 0.024). However, in the post-intervention evaluations, we did obtain significant differences between limited cervical rotation and some stabilometric variables, although fundamentally with weak and moderate values, which were stronger during follow-up. In the post-immediate evaluation, only weak correlations were obtained (ρ = 0.3–0.5; *p* < 0.05) with mean surface, the anterior and lateral COP displacements and mean COP-Y. On the 7th day, some weak correlations and some moderate correlations were obtained (ρ = 0.5–0.7; *p* < 0.001) with mean velocity, anterior COP displacement, mean path length, and mean surface. Finally, on the 15th day, in addition to some moderate correlations (e.g., with L/S), some strong correlations were obtained (ρ = 0.7–0.9; *p* < 0.001), with mean velocity, anterior COP displacement, and mean path length.

## 4. Discussion

As Coutler et al. has shown in a recent review [22], it is generally difficult to compare the manual therapy treatments (manipulation and mobilization) applied in patients with CNSNP, and its effects should be cautiously considered as unique techniques. A wide variety of techniques, usually with small samples, have been studied, which makes the current evidence heterogeneous. Due to this lack of consensus, it is difficult to compare the results due to the differences in the tests and variables measured in each study. In addition, a recent study emphasizes that the differences in the factors used to subclassify NSNP patients problematizes further comparison among studies; therefore, pain, mobility, and the association between them should be considered fundamentally for subclassifications [37].

The effects of manual therapy and, specifically, spinal HVLA manipulations on different musculoskeletal disorders have been studied. The effect of thoracic manipulations [49] or middle cervical manipulations on neck pain [50] is known, and even the beneficial effect of upper cervical HVLA manipulation on the range of motion, pain, and disability has been proven, as a single technique and together with other manipulations [24], mainly in patients with cervicogenic headache, cervical pain, and temporomandibular disorders [51].

However, as far as we know, there is no evidence yet about the effects of upper cervical manipulation on postural stability associated with the decreased range of motion of C1-C2 in patients with CNSNP.

Therefore, considering that cervical rotation affects standing postural control [35], and that the majority of cervical mobility occurs in C1-C2, particularly rotation [52], this would justify the therapeutic approach to upper cervical rotation dysfunctions with a HVLA manipulation in subjects with CNSNP that have an alteration of the postural control [9,53].

The purpose of this study was to show the effect of a single upper cervical spine (C1-C2) HVLA rotational thrust manipulation on CROM and standing postural control in patients with CNSNP, over 3 months or more, with a positive CFRT. We also wanted to observe the changes produced immediately after intervention and in a follow-up of 7 and 15 days.

### 4.1. Effects of Upper Cervical HVLA Manipulation on CROM

In our study, upper cervical rotation, after manipulation, showed significant differences from a positive CFTR, both immediately and during the 15 days of follow-up, always with values after manipulation of the dysfunctional side above 37.5° on the CFRT. Even despite the manipulation side, the left (group * time; *p* = 0.000; ƞ 2 *p* = 0.516) and right (group * time; *p* = 0.005; ƞ 2 *p* = 0.273) rotation increased significantly in the experimental group, between 3 and 4.5 degrees, and not in the control group, coinciding with the results of Clements et al. [54] on asymptomatic patients, where the asymmetry in rotations was reduced according to the CFRT, although with great variability in results between subjects, which is why they are data to consider with caution.

Our baseline data (lower than 32° on the CFRT) agree with other authors on C1-C2 mobility restriction for patients with cervicogenic headache [38,39,40,43,55], migraines [52,56], or neck pain [34]. However, they do not agree with other studies with values above 33° in low neck pain [38], migraines [39], or in asymptomatic patients [39,56].

According to Hall et al. [43], we must consider 7° for the CFRT as minimal detectable change (MDC) for patients with cervicogenic headache. In our results, we observed an increase of 7.41° in the post-immediate measurement, 6.82° on the 7th day, and 6.14° on the 15th day; therefore, we can consider them as clinically important. They are similar to those published by Malo-Urries et al. [57], with an increase in the CFRT of 7.3° after upper cervical translatoric mobilization, and also similar to those published by Dunning et al. [34], where they obtained a change between 5.9° and 8.4° with upper cervical and upper thoracic manipulation, versus non-thrust mobilization with smaller changes (between 2.5° and 3.5°). However, those last data with mobilization technique do not agree with those obtained by Mohamed et al. [55] with the SNAG rotation technique on C1-C2 and a 15.3° increase immediately after the intervention.

### 4.2. Effects of Upper Cervical HVLA Manipulation on Standing Postural Control

There are many factors that determine the measurements of the standing stability (number of trial recordings, duration, visual conditions, foot positions, surface conditions, and parameters measured), and it would be advisable to standardize them to facilitate comparability of results and reproducibility of procedures in future studies [44]. We know that patients with chronic neck pain have an impairment of postural control in comparison with healthy controls [53], even in young people with low disability and low intensity chronic idiopathic neck pain [58]. Therefore, the evaluation and treatment of postural stability in these patients is important, and, with our results, we agree to consider the clinical importance of evaluation and intervention on improving standing postural control. Nevertheless, in a recent cross-sectional study, differences between patients with chronic idiopathic neck pain and asymptomatic subjects were not found, and, in addition, the clinical utility of different cervical sensorimotor control tests was questioned [59].

Some studies have not observed changes in the proprioceptive system with different manual technique interventions on the cervical spine in nonspecific neck pain or cervicogenic dizziness [33,60,61]. However, in our study, we obtained very significant changes with the upper cervical spine HVLA manipulation on C1-C2 in the stabilometric parameters to assess postural control through standing stability, as well as a large effect size in all of them, agreeing with another study [62].

The accuracy of standing postural control improved and the effort to maintain the standing position (L/S values) decreased in the experimental group by 13.4% and 27.4% on the 7th and 15th day, respectively, with values approaching 1, which is the normal reference value in healthy adults [63]. Our baseline data on the surface length ratio L/S (1/mm) in patients with CNSNP were lower than those shown in other studies in patients with renal lithiasis [31] or in control subjects [36], and they were higher than those shown by other studies in healthy adults [63,64].

The mean velocity of the COP displacement progressively decreased from the intervention to the end of the study, reaching a reduction of up to 42.18% (from 2.11 ± 0.269 to 1.22 ± 0.199 m/s), which suggests a lower need for feed-back activity to rectify the position, according to Quek et al. [65]. However, the changes produced in the anterior and lateral mean velocity were not significant, although they showed in both cases a decrease in the experimental group compared to the control group. These data contrast with the lack of effect of manipulations on the mean velocity of the COP displacement in other studies [18,32,33].

The mean path length and the mean surface described by the displacement of the COP decreased considerably from the post-immediate measurement until the final of the follow-up, with decreases for the path length in the three post-intervention measurements (immediate, 7th, and 15th days) of 13.54%, 31.14%, and 42.06%, while, for the surface, it was 13.24%, from 22.29 % and 22.78%, which showed an important improvement in the standing postural control. This is the result of the decrease in the anterior (group * time; *p* = 0.000; ƞ 2 *p* = 0.706) and lateral (group * time; *p* = 0.000; ƞ 2 *p* = 0.511) displacements, with the greatest decrease in the antero-posterior axis.

Our baseline data on the mean surface of COP displacement in patients with CNSNP (Experimental Group (EG): 122.42 ± 25.679 mm^2^; control group (CG): 115.17 ± 15.807 mm^2^) are relatively low compared to other studies [17,18,59,66].

Furthermore, in general, the effect that we achieved on the displacements of COP variables after upper cervical spine HVLA manipulation is greater than in other studies, some of which showed no effect on postural sway after manipulation or other intervention, as with cervical spine manipulation in nonspecific neck pain [31], with sacroiliac manipulation [19] or lumbar spinal manipulation in low back pain [32], or with caudal talocrural joint manipulation in patients with ankle sprain [17].

The analysis of the global stabilometric variables of COP displacement showed significant differences during the follow-up. After reviewing the data with an overall view, we can see that patients were making changes in their posture during follow-up, obtaining the most significant results on the 15th day, without significant results in the immediately post-intervention measurement. The change in the accuracy factor and energy cost to maintain the standing position (L/S) was not significant in the immediate assessment, which tells us that just after manipulation, the patient does not improve the accuracy of postural control and the effort to maintain the standing position. This is the logical effect if we consider that we have applied a great stimulus to an especially important region for the postural system and it takes time to adapt to the immediate mechanical change in the ROM, measured with the CFRT. A significant improvement is observed in the L/S over time until the 15th day (*p* < 0.001), which is the period in which the postural system may have adapted to the new situation.

Quek et al. [65] considered that cervical flexion-rotation ROM asymmetry is an independent predictor of standing balance, over and above the influence of neck pain intensity. With our results, we can support this statement with some caution since, on one side, we obtained a similar behavior during the study follow-up in the values of the CFRT and the global stabilometric variables, but, on the other, we did not obtain a significant correlation between the baseline CFRT when it was positive, and the stabilometric values (*p* > 0.05). This makes us doubt the predictive value of CFRT for standing balance. Although, after the intervention, in the post-immediate measurements, but fundamentally on the 7th and on the 15th days, we obtained significant correlations between cervical rotation on the CFRT and some stabilometric variables of standing postural control, with strong correlations at the end of the study. This is something that should be studied in the future to clarify the relationship between functional impairment of the upper cervical spine and standing postural control.

### 4.3. Limitations

A limitation of the study presented is the sample size, based on the sample calculation from a pilot study and, although similar to many other studies, considering the recommendations, it is important to make an effort to carry out studies with a larger sample size, as well as prolong the follow-up. Another limitation is the use of a single HVLA technique, which always has greater difficulty in producing effects that can be extrapolated to clinical practice. In addition, the possible visceral etiology of neck pain and motion restriction was not controlled, so part of the sample might have received neck treatment instead of the right treatment for the visceral disorder. This fact might result in an incorrect estimation of the treatment’s effect size [67,68]. Another limitation could be the lack of control over pain intensity during study follow-up, although the initial aim of the study was not really to assess the influence of this factor. Finally, the lack of therapist blinding could be considered another limitation of the study.

Future studies could investigate the effect of upper cervical HVLA manipulation, whether associated or not with other clinically relevant interventions, trying to subclassify subjects with nonspecific neck pain [37], and assess postural standing stability and upper cervical movement under different conditions of origin, intensity and duration of pain. In addition, future studies should try to clarify the clinical relevance of postural analysis in subjects with nonspecific neck pain. Sterling et al. also hinted at the possible need to subclassify patients, but believed further research is required before treatment based on stratification can be recommended [28]. In addition, future research should try to clarify whether clinical relevance may be related to the evolution of pain and its medium and long-term correlation with the outcome variables of our study (cervical mobility and standing postural control). For this reason, future studies should also assess the results on pain and a longer evolution, not only in mobility and postural control, but also on pain.

## 5. Conclusions

As more is learned about sub-classifying patients with neck pain, the effects of different techniques and the underlying mechanisms of analyzing larger samples, as well as the design and adaptation of interventions, can be more effective. According to this randomized clinical trial, with its limitations, a single upper cervical spine (C1-C2) HVLA rotational thrust manipulation increases the rotational ROM in the upper cervical spine, especially on the restricted side, reducing the asymmetry and, at the same time, producing an improvement in standing postural control as measured by the variables of the COP displacements, due to amelioration accuracy and decrease in effort, thus the energy cost to maintain the standing position in patients with chronic nonspecific neck pain is reduced.

## Figures and Tables

**Table 1:** Descriptive baseline data of the experimental and control group. Means ± standard deviation are shown for continuous variables, and % (n) is shown for categorical variables.

Variable	Experimental	Control	*p*-Value
Gender, women %(n)	50.00 (11)	54.55 (10)	0.763
Age (year)	38.36 ± 11.664	37.50 ± 11.827	0.809
Weight (Kg)	64.32 ± 12.411	63.59 ± 9.404	0.828
Height (m)	1.67 ± 0.973	1.68 ± 0.626	0.971
BMI (kg/m^2^)	22.66 ± 3.349	22.55 ± 3.213	0.909
Limited Rotation (degrees)	31.36 ± 0.902	30.14 ± 1.167	<0.001 *
Mean velocity (mm/s)	2.11 ± 0.269	2.11 ± 0.280	0.996
Surface length ratio L/S (1/mm)	1.63 ± 0.455	1.67 ± 0.289	0.721
Mean Surface (mm^2^)	122.42 ± 25.679	115.17 ± 15.807	0.266
Mean Path length (mm)	189.20 ± 24.147	189.50 ± 25.256	0.998
Mean Pressure (g/cm^2^)	313.28 ± 51.123	299.65 ± 65.406	0.445
Mean COP X	−0.26 ± 5.328	0.60 ± 6.100	0.623
Mean COP Y	−5.36 ± 5.001	−3.67 ± 1.912	0.145
Mean Anterior Velocity (mm/s)	1.24 ± 0.366	1.16 ± 0.201	0.390
Mean Lateral Velocity (mm/s)	0.76 ± 0.181	0.85 ± 0.171	0.131

* Indicates significant differences comparing between groups (t test for independent variables); BMI: Body mass index; COP: Center of pressure.

**Table 2:** Inferential statistics analysis for the group factor and the interaction between factors (time * group) with two-way repeated measures ANOVA. Effect size was calculated as the partial eta squared (ƞ 2 *p*).

Variable	Group			Time * Group		
	F	*p*-Value	(ƞ 2 *p*)	F	*p*-Value	(ƞ 2 *p*)
Limited Rotation (degrees)	132.087 *	<0.001	0.759	46.447 *	<0.001	0.777
Surface length ratio L/S (1/mm)	6.133 *	0.017	0.127	16.434 *	<0.001	0.552
Mean velocity (mm/s)	41.636 *	<0.001	0.498	78.990 *	<0.001	0.856
Mean Surface (mm^2^)	4.040	0.051	0.088	14.695 *	<0.001	0.524
Mean Path length (mm)	41.668 *	<0.001	0.498	78.180 *	<0.001	0.854
Mean Pressure (g/cm^2^)	<0.001	0.999	<0.001	6.374 *	0.001	0.323
Mean COP X	0.559	0.459	0.013	0.668	0.576	0.048
Mean COP Y	16.046 *	<0.001	0.276	3.746 *	0.018	0.219
Mean Anterior Velocity (mm/s)	0.361	0.551	0.009	3.831 *	0.017	0.223
Mean Lateral Velocity (mm/s)	3.662	0.062	0.080	3.831 *	0.017	0.223

* Indicates significant differences; COP: Center of pressure.

**Table 3:** Interaction between factors (time * group) showing the differences by group at each evaluation (post hoc Sidak test).

Variable	Time-Evaluation	Differences (EG-CG)		*p*-Value	CI 95%	
		Mean	±SEM		Lower	Upper
Limited Rotation	Baseline	1.227 *	0.314	<0.001	0.593	1.862
(degrees)	Post-immediate	8.318 *	0.752	<0.001	6.800	9.836
	7 days	7.909 *	0.667	<0.001	6.563	9.255
	15 days	6.818 *	0.638	<0.001	5.530	8.106
Surface length ratio L/S	Baseline	−0.041	0.115	0.721	−0.273	0.190
(1/mm)	Post-immediate	−0.045	0.107	0.675	−0.262	0.171
	7 days	−0.235 *	0.073	0.002	−0.382	−0.089
	15 days	−0.460 *	0.065	<0.001	−0.591	−0.330
Mean velocity	Baseline	<0.001	0.083	0.996	−0.167	0.166
(mm/s)	Post-immediate	−0.271 *	0.077	0.001	−0.427	−0.116
	7 days	−0.645 *	0.072	<0.001	−0.791	−0.498
	15 days	−0.867 *	0.076	<0.001	−1.021	−0.713
Mean Surface	Baseline	7.246	6.429	0.266	−5.728	20.220
(mm2)	Post-immediate	−8.786	6.427	0.179	−21.757	4.184
	7 days	−20.845 *	5.535	0.001	−32.016	−9.675
	15 days	−21.023 *	5.276	<0.001	−31.670	−10.376
Mean Path length	Baseline	0.018	7.450	0.998	−15.016	15.052
(mm)	Post-immediate	−24.564 *	6.923	0.001	−38.536	−10.592
	7 days	−57.945 *	6.517	<0.001	−71.098	−44.793
	15 days	−78.023 *	6.892	<0.001	−91.932	−64.113
Mean Pressure	Baseline	13.638	17.699	0.445	−22.080	49.356
(g/cm2)	Post-immediate	−1.836	16.789	0.913	−35.717	32.045
	7 days	−5.280	16.415	0.749	−38.406	27.846
	15 days	−6.623	16.735	0.694	−40.394	27.149
Mean COP X	Baseline	−0.855	1.727	0.623	−4.339	2.630
	Post-immediate	−1.600	1.704	0.353	−5.039	1.839
	7 days	−1.255	1.578	0.431	−4.439	1.930
	15 days	−1.091	1.557	0.487	−4.232	2.051
Mean COP Y	Baseline	−1.695	1.143	0.145	−4.001	0.610
	Post-immediate	−3.477 *	0.958	0.001	−5.410	−1.545
	7 days	−4.259 *	0.874	<0.001	−6.022	−2.496
	15 days	−4.486 *	0.913	<0.001	−6.330	−2.643
Mean Anterior Velocity	Baseline	0.077	0.089	0.390	−0.102	0.257
(mm/s)	Post-immediate	−0.045	0.073	0.535	−0.192	0.101
	7 days	−0.118	0.068	0.088	−0.255	0.018
	15 days	−0.077	0.080	0.342	−0.240	0.085
Mean Lateral Velocity	Baseline	−0.082	0.053	0.131	−0.189	0.025
(mm/s)	Post-immediate	−0.145	0.076	0.062	−0.299	0.008
	7 days	−0.136	0.083	0.106	−0.303	0.030
	15 days	−0.155	0.078	0.053	−0.311	0.002

* Indicates significant differences; EG: Experimental Group; CG: Control Group; CI: Confidence interval; SEM: Standard error of the mean; COP: Center of pressure.

**Table 4:** Interaction between factors (time * group) showing the experimental group data differences in the pairs of means by the time factor (post hoc Sidak test).

Variable.	Time-Evaluation	Difference (Baseline-x)		*p*-Value	CI 95%	
		Mean	±SEM		Lower	Upper
Limited Rotation	Post-immediate	−7.409 *	0.463	<0.001	−8.687	−6.131
(degrees)	7 days	−6.818 *	0.411	<0.001	−7.952	−5.684
	15 days	−6.136 *	0.381	<0.001	−7.188	−5.085
Surface length ratio L/S	Post-immediate	0.007	0.058	1.000	−0.152	0.167
(1/mm)	7 days	0.219 *	0.064	0.008	0.042	0.395
	15 days	0.447 *	0.066	<0.001	0.265	0.629
Mean velocity	Post-immediate	0.283 *	0.046	<0.001	0.157	0.409
(mm/s)	7 days	0.655 *	0.041	<0.001	0.541	0.769
	15 days	0.884 *	0.040	<0.001	0.774	0.993
Mean Surface	Post-immediate	16.210 *	3.030	<0.001	7.845	24.575
(mm^2^)	7 days	27.287 *	2.989	<0.001	19.035	35.540
	15 days	27.892 *	3.605	<0.001	17.939	37.845
Mean Path length	Post-immediate	25.618 *	4.121	<0.001	14.240	36.996
(mm)	7 days	58.914 *	3.729	<0.001	48.618	69.210
	15 days	79.577 *	3.578	<0.001	69.698	89.456
Mean Pressure	Post-immediate	17.519 *	3.415	<0.001	8.090	26.948
(g/cm^2^)	7 days	18.817 *	3.388	<0.001	9.463	28.172
	15 days	19.873 *	3.226	<0.001	10.968	28.779
Mean COP X	Post-immediate	0.741	0.418	0.407	−0.412	1.894
	7 days	0.445	0.480	0.930	−0.880	1.771
	15 days	0.395	0.508	0.969	−1.007	1.798
Mean COP Y	Post-immediate	1.891 *	0.678	0.046	0.020	3.762
	7 days	2.814 *	0.695	0.001	0.895	4.732
	15 days	2.873 *	0.683	0.001	0.988	4.757
Mean Anterior Velocity	Post-immediate	0.164 *	0.058	0.043	0.004	0.324
(mm/s)	7 days	0.186 *	0.047	0.002	0.055	0.317
	15 days	0.150 *	0.052	0.037	0.006	0.294
Mean Lateral Velocity	Post-immediate	0.027	0.037	0.975	−0.074	0.128
(mm/s)	7 days	0.064	0.039	0.497	−0.043	0.171
	15 days	0.095 *	0.033	0.033	0.005	0.186

* Indicates significant differences; CI: Confidence interval; SEM: Standard error of the mean; COP: Center of pressure.
